# The Effects of a Simple Sensor Reorientation Procedure on Peak Tibial Accelerations during Running and Correlations with Ground Reaction Forces

**DOI:** 10.3390/s23136048

**Published:** 2023-06-30

**Authors:** Molly M. Bradach, Logan W. Gaudette, Adam S. Tenforde, Jereme Outerleys, José R. de Souza Júnior, Caleb D. Johnson

**Affiliations:** 1 Spaulding National Running Center, Department of Physical Medicine and Rehabilitation, Harvard Medical School, Cambridge, MA 02138, USA; 2 Department of Mechanical and Materials Engineering, Queen’s University, Kingston, ON K7L 3N9, Canada; 3 Faculty of Ceilandia, University of Brasilia, Brasilia 73340, Brazil; 4 Military Performance Division, United States Army Research Institute for Environmental Medicine, Natick, MA 01760, USA

**Keywords:** wearable sensors, inertial measurement units, IMU, gait

## Abstract

While some studies have found strong correlations between peak tibial accelerations (TAs) and early stance ground reaction forces (GRFs) during running, others have reported inconsistent results. One potential explanation for this is the lack of a standard orientation for the sensors used to collect TAs. Therefore, our aim was to test the effects of an established sensor reorientation method on peak Tas and their correlations with GRFs. Twenty-eight runners had TA and GRF data collected while they ran at a self-selected speed on an instrumented treadmill. Tibial accelerations were reoriented to a body-fixed frame using a simple calibration trial involving quiet standing and kicking. The results showed significant differences between raw and reoriented peak TAs (*p* < 0.01) for all directions except for the posterior (*p* = 0.48). The medial and lateral peaks were higher (+0.9–1.3 g), while the vertical and anterior were lower (−0.5–1.6 g) for reoriented vs. raw accelerations. Correlations with GRF measures were generally higher for reoriented TAs, although these differences were fairly small (Δr^2^ = 0.04–0.07) except for lateral peaks (Δr^2^ = 0.18). While contingent on the position of the IMU on the tibia used in our study, our results first showed systematic differences between reoriented and raw peak accelerations. However, we did not find major improvements in correlations with GRF measures for the reorientation method. This method may still hold promise for further investigation and development, given that consistent increases in correlations were found.

## 1. Introduction

Early stance ground reaction forces (GRFs) during running have been of interest to researchers and clinicians due to their associations with running-related musculoskeletal injuries [1,2,3]. While GRFs can be measured using an instrumented treadmill in laboratory settings, tibial accelerations (TAs) have gained popularity due to their correlation with early stance GRFs and their relative ease of collection [4]. Peak vertical and resultant TAs have been correlated with vertical GRF loading rates, the metric most consistently associated with injury, as well as peak vertical forces [5,6,7,8,9]. In addition, peak TAs and loading rates in the posterior and medial-lateral directions have shown strong associations, albeit with more limited evidence [10]. In relation to the ease of measurement, peak TAs can be captured using small, lightweight, and wireless wearable sensors, making it an ideal measure for clinic or field environments. 

While early work has shown promise for the use of peak TAs as a surrogate for early stance GRFs during running, there has been considerable variation in the reported correlations between these two measures. Focusing on vertical GRF loading rates, some studies have reported strong to excellent correlations with peak vertical TAs (r > 0.8) [5,6,7]. However, other studies have found much lower correlations, ranging from weak to moderate (r = 0.47–0.66) [8,9]. In one study by Van den Berghe et al. [5], the reported correlations even varied significantly by running speed condition, with coefficients ranging from 0.64 to 0.84. One possible explanation for this inconsistency in reported correlations could lie in the orientation of the sensors used to collect TAs.

**Figure 1:**
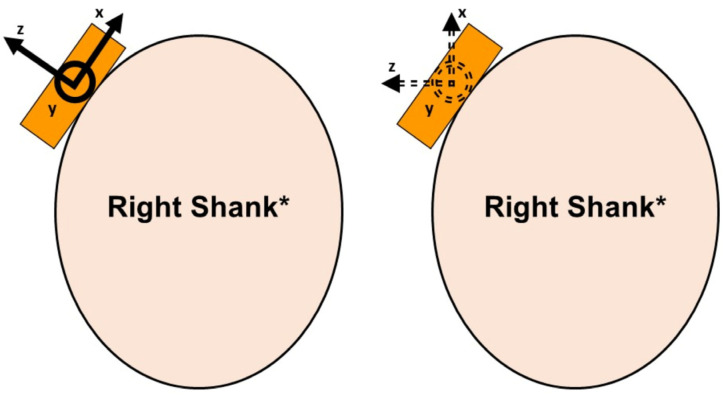
Example of misalignment of accelerometer axes with sensor-fixed frame (**left**) and potential correction with reorientation (**right**). * Top view, or cross-section, of shank.

Without calibration into a global reference frame, the axes of an accelerometer are in a sensor-fixed orientation (i.e., relative to whatever orientation the sensor is attached to a body with). With standard attachment methods for collecting TAs, this orientation tends to be rotated out of a plane relative to the GRF due to the anatomy of the distal-medial tibia (Figure 1). Further, the degree and nature of the misalignment could vary according to how the sensor is attached to the tibia and individual anatomy. Cain et al. [11] described a method for reorienting the axes of an accelerometer into a body-fixed frame or relative to the path of motion of the limb that the sensor is attached to (Figure 1). In theory, this could align the accelerations more closely to the GRF planes, potentially yielding better correlations between the two measures. Further, this reorientation method is simple and quick, making it easy to incorporate into clinic/field environments. 

While this reorientation method has good construct validity [11], no previous work has demonstrated its effects on resulting TAs during running. Therefore, the purpose of this study was to establish the effects of reorienting the sensor axes to a body-fixed frame on peak TAs during running, as well as the relationships between peak TAs and early stance GRFs. Our primary hypotheses were that the sensor reorientation procedure would systematically change peak TAs and improve the correlations between peak TAs and early stance GRF metrics.

## 2. Materials and Methods

Data from 28 runners (13 males, 15 females) undergoing treatment at a running clinic were utilized for this study (Mean age = 39 ± 13 years, height = 1.72 ± 0.09 m, mass = 68.5 ± 10.7 kg). Runners underwent a standard biomechanical assessment as part of their treatment, involving running at a self-selected speed on a treadmill. All participants were able to run for at least 10–15 min at the time of assessment. This study was conducted according to the guidelines of the Declaration of Helsinki and approved by the Institutional Review Board (or Ethics Committee) of Mass General Brigham (Protocol #2017P000481, 07/17/17). A waiver of informed consent was obtained from the board for the secondary use of data that were collected as part of a patient’s standard of care. 

**Figure 2:**
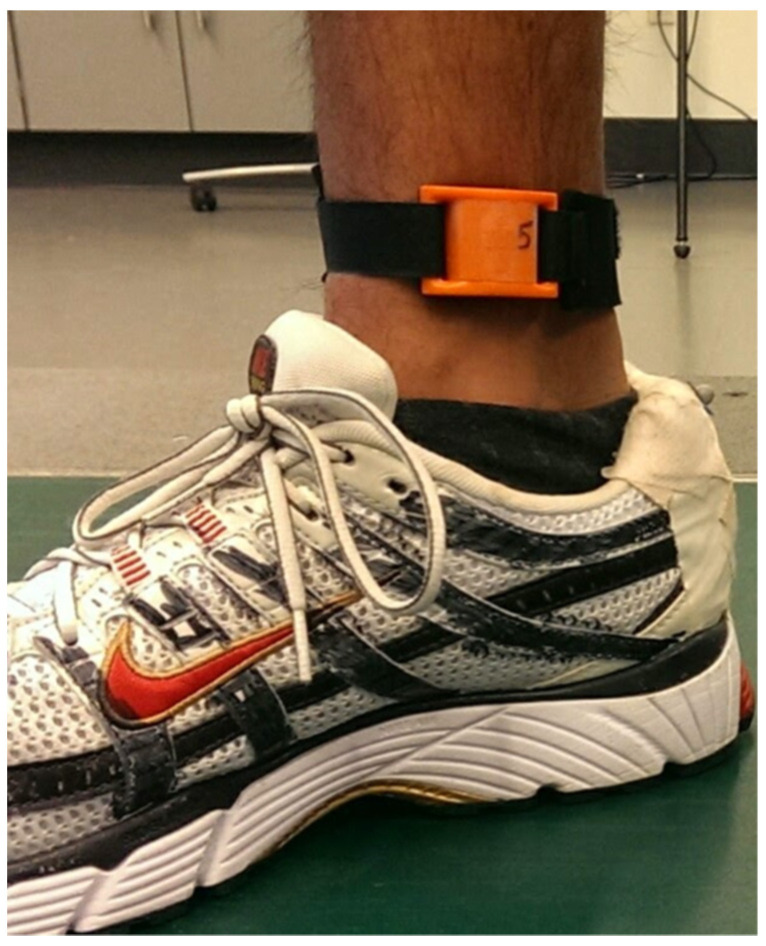
Attachment of inertial measurement unit on distal medial tibia.

Runners wore an inertial measurement unit (IMeasureU Blue Thunder, Auckland, New Zealand; weight = 12 g; accelerometer: range = ±16 g, resolution = 16-bit; gyroscope: range = ±2000°/s, resolution= 16-bit). The sensor was strapped to their right distal medial tibia, approximately one centimeter above the medial malleolus (Figure 2). The positive direction for each axis was: x = medial, y = anterior, and z = vertical. Participants completed a calibration trial involving a three-second period of quiet standing, followed by lifting their right leg and flexing/extending at the knee five times. During the calibration trial, the accelerometer and gyroscope of the inertial measurement unit were sampled at 500 Hz. Next, participants ran on an instrumented treadmill (AMTI, Watertown, MA, USA) embedded with two force plates (sampling rate = 1500 Hz) at a self-selected speed (mean speed = 2.81 ± 0.39 m/s). Participants were given a 3-min warmup, after which 16 s of data were collected, allowing for at least 20 strides of data. During the run, only accelerometer data were sampled from the inertial measurement unit at 1000 Hz.

All data processing was conducted using custom MATLAB scripts (Natick, MA, USA). Data from the calibration trial (accelerometer and gyroscope data included) were used to create a reorientation matrix, as described previously [7,11]. From the period of quiet standing, the vertical axis was defined in line with gravity. The medial-lateral axis was defined using gyroscope data from the period of the kicking movement as the average axis of rotation during knee flexion extension. This was performed using principal components analysis, assuming the unit vector from the first principal component to be the average axis of rotation. Finally, the cross product of these segments defined the anterior–posterior axis. The matrix was used to reorient accelerometer data from the running trial. 

Medial-lateral and anterior-posterior accelerations were low-pass filtered using a 4th-order bidirectional Butterworth filter (cutoff = 60 Hz) to help with peak identification [12]. The peak accelerations for each stride were extracted for all three axes using previously described methods [7,10]. The peaks were identified for both raw and reoriented accelerations. For the vertical axis, only peak positive accelerations were extracted. For the remaining axes, peak positive (medial/anterior) and negative (lateral/posterior) accelerations were extracted and expressed as an absolute value for simplicity.

Ground reaction forces were filtered using a 4th-order, low-pass, bidirectional Butterworth filter (cutoff = 50 Hz). Stance phase was identified for each stride using a vertical force threshold of 10 N. Vertical instantaneous loading rates (VILR) were calculated as the peak change in the vertical forces from initial contact to one of two endpoints: the peak vertical force or a transient impact peak force, when one was present [13]. Medial-lateral instantaneous loading rates (MILR/LILR) were calculated during the first 25% of the stance, and posterior instantaneous loading rates (PILR) during the first 15% [14]. Anterior loading rates were not considered, as the dominant anteriorly directed force occurrs during the late stance phase in running. Variables (TA and GRF) were averaged across all available strides for each participant.

Statistical analyses were performed in SPSS (IBM, Armonk, NY, USA). Normality was assessed using Shapiro-Wilk tests. The mean differences between raw and reoriented peak TAs were tested for using dependent *t*-tests or Wilcoxon signed-rank tests for non-parametric variables. Cohen’s d was calculated to assess the effect sizes. Correlations between peak TAs and early stance GRF metrics were assessed using Pearson’s Correlation coefficients or Spearman Rank coefficients for non-parametric variables. Outliers and influential points were assessed using scatter plots, and in the case of a suspected outlier, sensitivity analyses were run by removing outliers and recalculating correlations. If the outliers were found to have a significant effect on the results, they were excluded (only for the analysis in question). The difference in the coefficient of determination (r^2^) between correlations for raw and reoriented TAs was calculated to assess the practical significance of differences in these correlations. The alpha level for significance was set at 0.05.

## 3. Results

**Figure 3:**
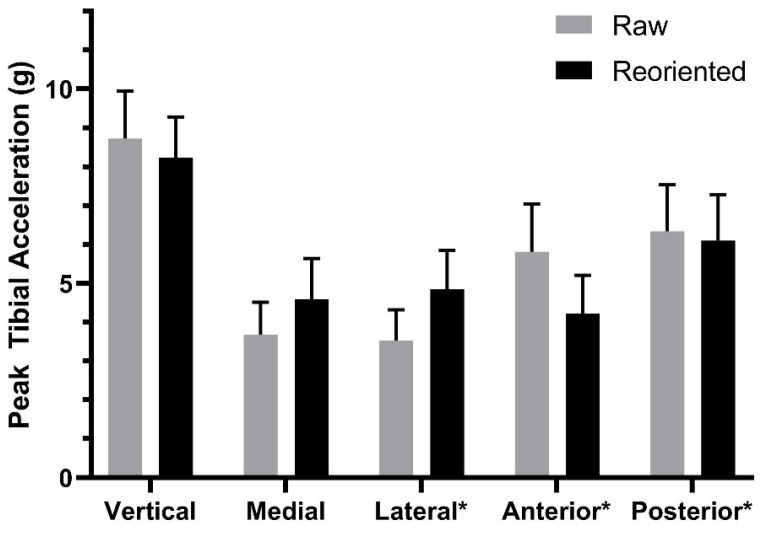
Mean differences between raw and reoriented peak tibial accelerations. All differences were significant at *p* < 0.01 except for posterior tibial accelerations. * Denotes results of Wilcoxon signed-rank test; all others are result of dependent *t*-tests.

Descriptive statistics are presented for GRF variables in Table 1, and the results of the tests of mean differences between the raw and reoriented peak TAs are shown in Figure 3. With accelerations reoriented, we found significantly higher medial (mean difference = 0.91 g, *p* < 0.01, d = 0.61) and lateral (mean difference = 1.31 g, *p* < 0.01, d = 0.74) peak TAs compared to the raw accelerations. However, we found significantly lower vertical (mean difference = −0.49 g, *p* < 0.01, d = 0.68) and anterior (mean difference = −1.6 g, *p* < 0.01, d = 0.79) peak values. There were no significant differences in posterior peaks (*p* = 0.48).

The results of correlation analyses between peak TAs and GRFs are found in Table 2. Overall, the correlations were stronger for reoriented TAs compared to raw, with differences in r^2^ ranging from 0.04 to 0.18. The largest difference was for lateral peak TAs and LILR, showing 18% more variance explained by the reoriented TAs. Only correlations between peak medial TAs and MILR were lower for reoriented accelerations, with a difference in r^2^ of −0.02.

## 4. Discussion

The purpose of this study was to establish the effects of a simple sensor reorientation procedure on resulting peak TAs during running and their correlations with important early stance GRF metrics. Our first finding was that there were significant differences in peak TAs after the accelerometer axes were reoriented into a body-fixed frame, confirming our first hypothesis. The direction of these differences varied by axis, with higher peaks in the medial-lateral directions and lower in the vertical and anterior. These findings were intuitive given that the accelerometer axes were orthogonal; if the peaks in one axis were increased by the reorientation, then it was likely that peaks along other axes would be reduced. 

As mentioned previously, the natural position of the sensor over the distal-medial tibia leads to the medial–lateral axis being rotated in the transverse plane, with the medial pointing more anteriorly. Therefore, our results could suggest that with this orientation, some of the acceleration that was in the true medial-lateral direction was recorded, or “lost”, in the anterior–posterior axis. One important limitation to our results, however, is that we did not have a ground truth for TAs. Given the time and logistical constraints of collecting data on patients, we were not able to collect either bone-mounted accelerometer data or, more realistically, three-dimensional kinematics to estimate a ground truth for TAs. While it is likely that the reoriented accelerations were closer to the true accelerations of the tibia, given the construct validity of the reorientation procedure, we could not verify this. This is a possible direction for future research. 

Our second primary finding was that correlations were generally improved between peak TAs and corresponding early stance GRF variables after their reorientation, partially confirming our hypothesis. However, these differences were small for vertical and posterior loading rates (+4–7% variance explained), and correlations with medial GRF loading rates were slightly lower after reorientation (−2% variance explained). Only lateral peak TAs showed practically significant improvements in correlation, with an additional 18% of variance explained in the loading rates after reorientation. It is also worth noting that correlations for vertical TAs were still slightly lower, even after reorientation, than in previously mentioned studies that found strong correlations (r > 0.8) [5,6,7].

It is difficult to explain why systematic changes in peak TAs were observed, but larger improvements in correlations with GRFs were not. One explanation may be that other factors, such as the technical specifications of the sensor and how tightly it was attached, matter more than the orientation of the sensor on the leg. In support of this, two studies found stronger correlations for vertical TAs utilizing sensors with a higher operating range (24–200 g), and one utilized a higher sampling rate (1600 Hz) than in the current study [5,7]. Additionally, both of these were laboratory studies where the tightness of the sensor attached to the tibia could be better controlled than in the current study, which was conducted in a clinic. One previous study on the correlations between TAs and GRFs was also conducted in a clinic environment and found similar correlations to ours (r = 0.66) [8].

A second explanation for the nominal improvements in correlations with reorientation may lie in the nature of the sensor reorientation procedure used. We chose to investigate this procedure for its simplicity, allowing for easy implementation in clinic and field environments. However, the procedure rotated the TAs into a body-fixed frame relative to the path of motion for the tibia. While this should be an improvement, it does not perfectly align the accelerometer axes with GRFs, which are in the reference frame of the treadmill. For instance, during the early stance in running, the tibia was rotated in the sagittal plane, with the proximal head posterior to the distal [15]. Therefore, when reoriented to the path of motion of the tibia, peak TAs would still be slightly misaligned relative to the GRF reference frame. There may also be individual variability in the alignment of the tibia due to the movement of the leg in the transverse and frontal planes, although movement in these planes is smaller than in the sagittal plane. It is fairly easy with modern motion capture systems to transform the coordinate systems of an accelerometer into that of a force plate. Therefore, one possible direction for future research may be to identify whether a reorientation procedure can be developed that adjusts the accelerometer axes dynamically throughout the stance based on the rotation of the tibia to match the GRF coordinate system. This may be possible by utilizing data from the gyroscope; however, it is worth considering the effects on onboard storage space and battery life that would be required to accommodate sampling from an additional sensor. This is especially important when considering clinic or field applications, where recording sessions may last for over an hour [16,17].

The primary limitation of the current study was that the correlations between two measures do not necessarily mean that the measures will show the same predictive value for musculoskeletal injury. Therefore, demonstrating the correlations between peak TAs and GRF measures that have been associated with injury provides a good basis for their potential as a screening or tracking tool for injury risk. However, there is a need for more research to establish that peak TAs, particularly in the field, relate directly to musculoskeletal injuries in runners. There is some evidence for this [16,18], but it has been very limited to date.

## 5. Conclusions

This study was the first to examine the effects of a simple reorientation procedure on peak TAs and correlations with GRF measures. We found that the reorientation procedure had a significant mean effect on peak TAs, suggesting that it at least partially corrected accelerations for the misalignment of the sensor axes with the true path of motion in the tibia. However, while small improvements were found for the vertical and posterior axes, the only practically significant improvements in correlations found were for lateral TAs and GRFs. This suggests that for clinical practice, where peak TAs were generally used as a surrogate for external lower extremity loads (i.e., GRFs), the reorientation procedure does not have a significant effect in its current state. Further investigation and development could improve these results, potentially by developing a dynamic reorientation procedure that adjusts TAs differently depending on the orientation of the tibia.

## Figures and Tables

**Table 1:** Descriptive statistics for ground reaction force variables.

	Mean (SD)	95% Confidence Interval
**VILR (BW/s)**	75.3 (23.7)	66.1–84.5
**MILR (BW/s)**	8.9 (4.0)	7.4–10.4
**LILR (BW/s)**	11.0 (7.7)	8.0–14.0
**PILR (BW/s)**	8.9 (3.9)	7.4–10.4

SD, standard deviation; VILR/MILR/LILR/PILR, vertical/medial/lateral/posterior instantaneous loading rate.

**Table 2:** Results of correlation analyses between peak tibial accelerations and ground reaction forces.

	Raw	*p*	Reoriented	*p*	Δr^2^
**Vertical TA—VILR**	0.618 0.308–0.801	<0.01	0.647 0.351–0.818	<0.01	0.04
**Medial TA—MILR**	0.808 0.609–0.906	<0.01	0.795 0.578–0.901	<0.01	−0.02
Lateral TA—LILR *	0.761 0.528–0.888	<0.01	0.871 0.725–0.942	<0.01	0.18
Posterior TA—PILR *	0.568 0.228–0.784	<0.01	0.628 0.298–0.824	<0.01	0.07

All results are presented as: coefficient—95% confidence interval. * Denotes Spearman Rank coefficient; all others are Pearson’s Correlation coefficients. For Spearman Rank coefficients, confidence intervals were estimated using Fisher’s r to z transformation and standard errors based on the formula proposed by Fieller, Hartley, and Pearson. VILR/MILR/LILR/PILR, vertical/medial/lateral/posterior instantaneous loading rate.

## Data Availability

The data for this study are not publicly available due to them being part of the patient’s protected medical information.
